# Are trait-growth models transferable? Predicting multi-species growth trajectories between ecosystems using plant functional traits

**DOI:** 10.1371/journal.pone.0176959

**Published:** 2017-05-09

**Authors:** Freya M. Thomas, Peter A. Vesk

**Affiliations:** School of BioSciences, ARC Centre of Excellence for Environmental Decisions, The University of Melbourne, Victoria, Australia; Chinese Academy of Forestry, CHINA

## Abstract

Plant functional traits are increasingly used to generalize across species, however few examples exist of predictions from trait-based models being evaluated in new species or new places. Can we use functional traits to predict growth of unknown species in different areas? We used three independently collected datasets, each containing data on heights of individuals from non-resprouting species over a chronosquence of time-since-fire sites from three ecosystems in south-eastern Australia. We examined the influence of specific leaf area, woody density, seed size and leaf nitrogen content on three aspects of plant growth; maximum relative growth rate, age at maximum growth and asymptotic height. We tested our capacity to perform out-of-sample prediction of growth trajectories between ecosystems using species functional traits. We found strong trait-growth relationships in one of the datasets; whereby species with low SLA achieved the greatest asymptotic heights, species with high leaf-nitrogen content achieved relatively fast growth rates, and species with low seed mass reached their time of maximum growth early. However these same growth-trait relationships did not hold across the two other datasets, making accurate prediction from one dataset to another unachievable. We believe there is evidence to suggest that growth trajectories themselves may be fundamentally different between ecosystems and that trait-height-growth relationships may change over environmental gradients.

## Introduction

Ecologists struggle to make predictions. Those we do make, often speak to patterns over very long time periods, or refer to very specific questions constrained to the scope of local datasets [1]. Making sensible predictions from limited empirical datasets to new situations, on time-scales relevant for management decisions is a difficult task [2]. There is a wide interest in testing whether it is feasible to predict ecosystem dynamics through the use of plant functional traits [3, 4]. Can easily measured traits help to reveal patterns that can be generalized over broad suits of species? The answer to this question remains unresolved, despite a broad literature motivated to finding general relationships between traits and environmental change [5–8], disturbance [9–14] and demographic rates such as growth [11, 15–26].

Improved predictive power might be achieved by measuring functional traits at the same time in the same location [24], by being cautious of using traits drawn from databases for inference at a local scale [27, 28] and by having a more local focus instead of searching for ‘monolithic global relationships’ [4]. These points may be particularly relevant to making local-scale trait based predictions, as we know that functional traits shift over productivity and climatic gradients [6, 29–33], intraspecific trait variability increases with sampled geographic range [27], leaf traits and wood density are known to vary up to three fold within species due to differences in light [18 and references therein], and the strength and shape of the successional shift of functional traits changes among eco-regions [34] and ontogeny [35, 36]. Therefore some broad trade-offs between traits may not be captured locally.

Gaining greater predictive capacity in trait-based ecology may also require stronger quantitative tests of model transferability, which is a hard test of how general a model actually is. Whilst attention to model transferability has increased within the species distribution modeling literature [37,38] it is less common in other ecological modeling [39,40]. The extent to which local environmental effects confound generalization of trait-based predictions can be tested through out-of-sample predictions that aim to assess model transferability. This is the aim of our study; testing the transferability of trait-based multi-species hierarchical models between different environments, species, and trait distributions.

We tested trait-height-growth relationships by examining the influence of specific leaf area, woody density, seed size, and leaf nitrogen content on three aspects of plant growth: maximum growth rate, age at maximum growth, and asymptotic height. We used three independent datasets of plant height growth over time from different ecosystems to first compare how general trait-growth relationships are across different environments and second, to test the transferability of our trait-based models. We do this by predicting species growth trajectories from traits to out-of-sample data, a form of model validation rarely performed in ecological literature [40]. Last, modeling of all the datasets combined together did not increase our predictive ability but did reveal an extra dimension of growth variation not apparent within the independent datasets. By testing our capacity to perform out-of-sample prediction of growth trajectories between ecosystems using species functional traits, we found evidence to suggest that these trajectories may be fundamentally different between ecosystems and that therefore trait-height-growth relationships may change over broad environmental gradients.

## Materials and methods

### Datasets

**Fig 1:**
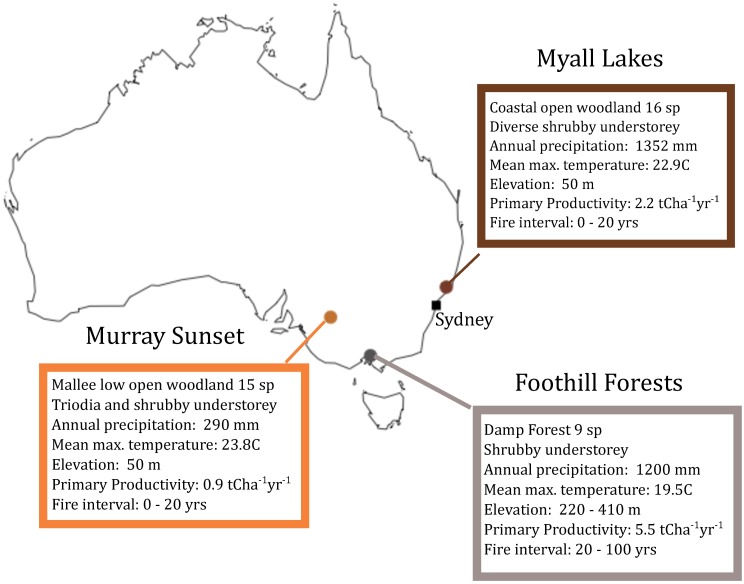
Study site locations, species sample sizes and environmental attributes.

We used three independently collected datasets, each containing data on heights of individuals from non-resprouting species over a chronosquence of time-since-fire sites from three ecosystems in south-eastern Australia, as well as functional traits which were collected for each species at each site following established protocols [41]. Murray Sunset is an area of low, open woodland within the semi-arid Murray Mallee region (34.7683° S, 141.8542° E) (field permit from The Department of Environment, Land, Water and Planning), Myall Lakes Region (32.4541° S, 152.3404° E) is an open sclerophyll forest region, and the Foothill Forest region (37.8700° S, 145.3539° E) is an area of damp open lowland shrubby forests (Fig 1)[42]. For details on sampling methodology see [43,44] and S1 File, see Fig 1 for sample sizes and study site locations.

### Hierarchical, non-linear growth models

We built a hierarchical, non-linear growth model, with two components.

An observation model (Eq 1) treats the heights of individual plants as drawn from a lognormal distribution. The lognormal distribution reflects the natural constraints of height data, which are only positive numbers, with relatively few extreme height values [45]:

$${\text{H}}_{i}\sim \;lnorm\left(\mu_{i},\sigma^{-2}\right),$$ (1)

where H is the observed height (cm) for each individual *i* which is drawn from a lognormal distribution with mean *μ*_*i*_ and precision *σ*^−2^.

The statistical process model modeled individual heights *μ*_*i*_ with a nonlinear growth model. A range of possible growth models exist [46–48]. We chose a sigmoidal model referred to as the Hillslope equation [49], which is a re-paramaterisation of a logistic equation with three parameters (Eq 2):

$$\mu_{i,j}=\frac{\alpha_{j}}{1+\text{exp}\left[-b_{j}\left(T_{i}\;-c_{j}\right)\right]}.$$ (2)

Here *μ*_*i*, *j*_ is the expected height of individual plant *i* of species *j* (cm) and *T*_*i*_ is the observed time since fire (or age in years) of each individual *i*, and *α*, b, and c are species-specific constants. These are biologically interpretable: *α* is the maximum height achieved (cm), the slope (b) represents the maximum annual relative growth rate (cm cm^-1^ yr^-1^), and c is the time at which this maximum growth occurs (yrs) (the location parameter). These growth parameters were each modeled as drawn from normal distributions with hyperparameters for the grand means and between species standard deviations estimated from the data.

We explored the relationships between parameter b, the parameter relating to growth rate and two other derived measures of growth rate, absolute growth rate and relative growth rate (S3 Fig). We found that there was a tight relationship between the Hillslope parameter of growth rate and relative growth rate (R^2^ between 0.73 and 0.99) and these were also correlated to measures of absolute growth rate. We also explored the correlations between these measurements of growth rate and functional traits (S4–S6 Figs).

### Including functional traits as species-specific linear predictors of growth

We used functional traits as predictors of the growth parameters in the growth model [49]. We begin by incorporating functional traits into one of our datasets only. We used the Myall Lakes dataset because this area has a mix of tall and short species, which represents a midpoint between the low growing Murray Sunset species and the tall growing Foothill Forests species. Each of the three parameters in our process model of growth were estimated via parameter sub-models, Eqs 3–5. Mean parameter values are estimated by linear models, with species trait values as covariates and intercepts also varying by species.

In deciding which functional traits to include in our models we relied on literature to generate hypotheses of trait effects on growth. We also wanted to minimise the numbers of parameters, to avoid over-parameterization. We began by considering that seed mass should be most influential on initial growth [50], stem density on achievable height [18,19,43,51] and leaf traits potentially throughout the whole growth process [20,37]. In addition to this, we calculated the Pearson’s R^2^ coefficient of determination between observed and predicted height values in a model where we let all traits influence each parameter in our growth model (R^2^ = 0.8751) and then used a process of backwards selection to test different trait-parameter combinations. Our final model had SLA influencing maximum height, seed mass on age at maximum growth and leaf nitrogen content on maximum relative growth rate (see Eqs 3–5) and had almost the same R^2^ (0.8752), and so we chose to use the model with fewer parameters:

$$a_{j}=\text{exp}\left(\beta 0+\;\beta 1\;\times {\text{ SLA}}_{j}+\;E_{j}\right),$$ (3)

$$b_{j}=\text{exp}\left(\beta 0+\;\beta 1\;\times \;N_{j}+\;E_{j}\right),$$ (4)

$$c_{j}=\text{exp}\left(\beta 0+\;\beta 1\;\;\times \;\;{\text{SM}}_{j}+\;E_{j}\right).$$ (5)

Where independent *ε* terms represent species level random effects and describe the residual variation between species in the predictive parameters after trait effects.

We used Bayesian inference, employing Markov chain Monte Carlo (MCMC) methods with the open-source software package JAGS version 3.3.0 [52] run via the statistical software environment R version 2.15.2 (R Development Core Team 2015) with the package R2jags [53]. Priors for mean parameters were normally distributed, centered on zero with a standard deviation of 0.0001. We used weakly informative priors for the observation error and random effects. These standard deviations were modeled as positive half Cauchy distributions, with prior mean 0 and prior scale 25 [54]. We initialized our model by using mean parameter values taken from single species non-linear models.

### Model convergence and checking

Three chains were monitored to ensure convergence, which was assessed both visually and via the Brooks-Gelman-Rubin convergence diagnostic [55]. We evaluated model fit through checks of predicted versus observed heights and by comparing Deviance Information Criteria (DIC)[56] between alternative models. We evaluated the fit of our species-specific linear sub-models by calculating Gelman’s R^2^ statistic (this R^2^ statistic can be interpreted like a classical coefficient of determination; it reports the proportion of variance in the response explained by the predictors for each level of a multi-level model) and pooling factors (which assesse the contribution of mean model group estimates versus data) for each sub-model [57]. We plotted coefficient estimates with 95% credible intervals of the linear predictors in our growth parameter sub-models. An approximate hypothesis test can be made by comparing the overlap of two 95% CIs. If they overlap by less than half their length then the null hypothesis of no difference can be rejected with p<0.05. If the intervals just touch, the null hypothesis can be rejected with p<0.01 [58]. However, a more relevant inference is about the consistency or differences between model coefficients, which we focus our discussion on.

### Predicting growth trajectories between datasets

Our hierarchical trait-based model of the Myall Lakes data reveals how the functional traits influenced each of the three growth parameters. We used these trait-growth relationships to undertake out-of-sample prediction to the species in the other two datasets based on their trait information only. To do this, we took the species trait data from Murray Sunset National Park and centered and scaled it based on the Myall Lakes trait data. We then predicted heights of Murray Sunset species, based on their trait values but using the trait-growth relationships determined in our Myall Lakes trait model. In this way, we could predict the growth trajectories of species based only on their trait data, using a trait growth model that was trained on the independent Myall Lakes dataset. We predicted the growth trajectories of each of the Murray Sunset and Foothill forest species based on our Myall Lakes model. We made these predictions within the model framework, by adding each species we wanted to predict to within the Myall Lakes dataset but with missing height values but corresponding trait values for each missing height data point. Using Bayesian methods, these missing values are imputed by Monte Carlo simulation.

### Evaluating predictions

We measured the accuracy and bias of our trait-based predicted growth trajectories with error statistics. Root mean squared deviance (RMSD) is the square root of the mean of the squared differences between observation and prediction. It quantifies the average distance of repeated samples from a known truth and is a measure of accuracy, the lower the value of RMSD, the closer the simulation is to the measurement [59,60]. Bias is measured as the mean deviance (MD) and reports the direction and size of the difference between the mean prediction and observation [59]. We use these error statistics to compare trait-based predictions to those of a multi-species model with no traits.

### Trait-relationship checking with independent datasets and combined datasets

To test whether the same trait relationships might hold between datasets, we took our trait based model constructed with the Myall Lakes dataset and used the same model and trait structure to train separate models for both Murray Sunset and Foothills datasets. As for the original trait models fitted to the Myall Lakes dataset, we assessed the R^2^ statistics between models fitted with all traits influencing all parameters (Murray Sunset 0.785 and Foothills 0.793) and with the smaller trait parameter set with the same structure that was used for Myall Lakes (Murray Sunset 0.779 and Foothills 0.791). Once again, there was little difference between these values.

To test whether a combined dataset, which increases the number of species and may increase the range of trait values, strengthens trait-growth parameter relationships, we then combined all three datasets. To account for site-specific differences in this model we allowed the intercept for each growth parameter to vary by system but this did not considerably improve the model (S2 Fig).

## Results

**Fig 2:**
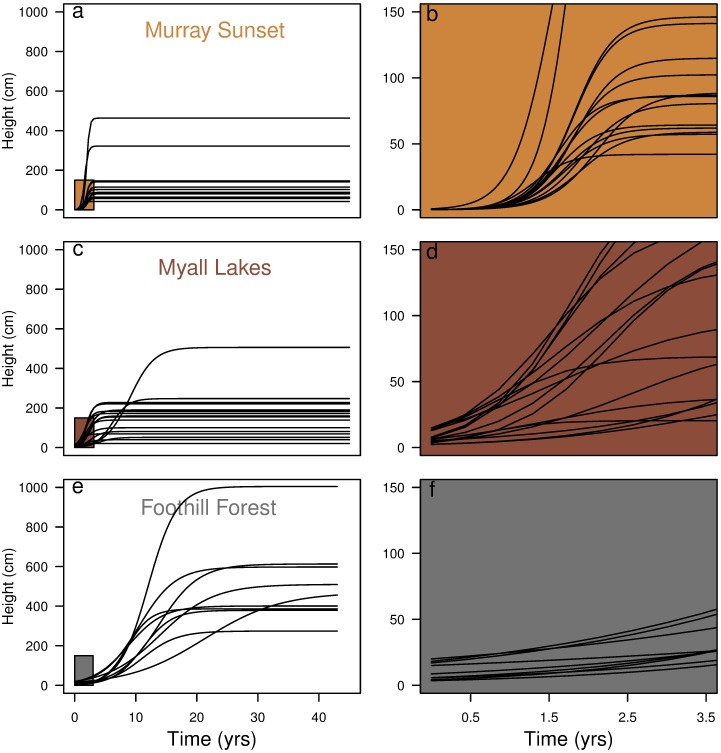
Growth trajectories of species from a hierarchical model without traits, fitted independently to each dataset. Each ecosystem had species with varied growth, but these differed considerably between systems; Murray Sunset species (a-b) grow quickly relative to Myall Lakes species (c-d), and Foothill Forest species (e-f) are all relatively tall and slow growing compared to the species in the two other ecosystems.

The species within these three datasets displayed broad differences between their growth patterns (Fig 2). Murray Sunset species had the fastest maximum relative growth rate, reached their maximum growth rates earlier and achieved the shortest heights compared to species in the other two datasets (Fig 2a). The opposite extreme is the Foothills forest, which had the slowest growing species, which reached their maximum growth rates latest and achieved the tallest heights of all the species between each of the ecosystems (Fig 2c). Myall Lakes had a mixture of tall and short species, which had maximum growth rates ranging from slow to fast. Myall Lakes also had the greatest temporal overlap in species dominance (Fig 2b).

**Fig 3:**
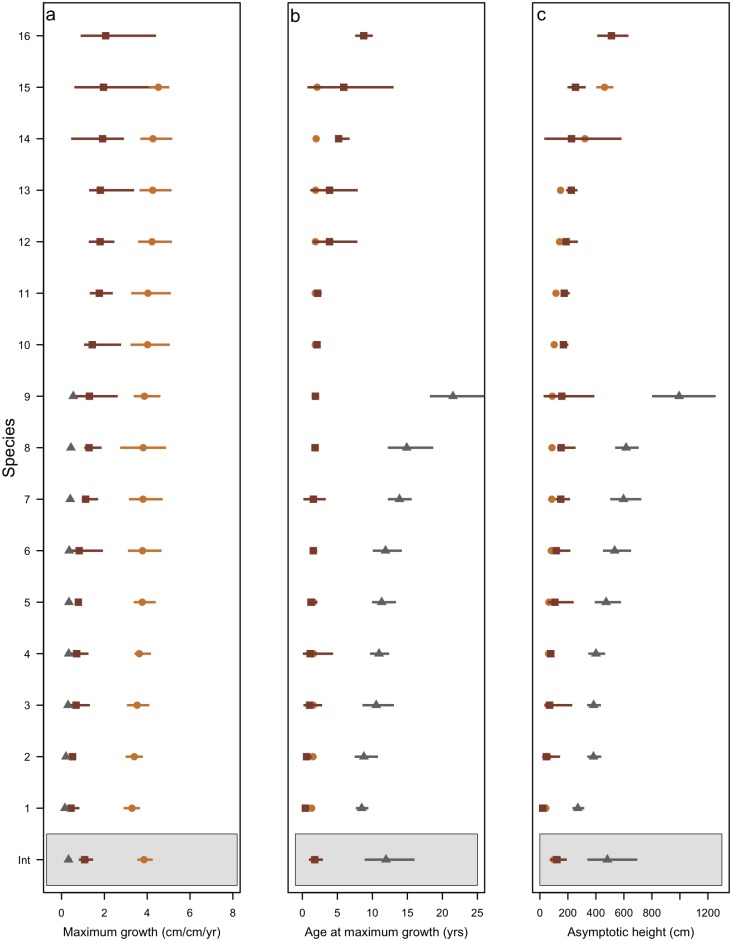
Species estimates for the three growth parameters for each dataset, with Murray Sunset species in yellow, Myall Lakes in brown and Foothill forests in black; a) maximum relative growth rate, b) age at maximum growth and c) asymptotic height. Bars indicate standard deviations around modeled parameters. The shaded panels at the base of each figure show the intercepts of the three separate hierarchical models, which are the mean parameter values.

Maximum relative growth rates varied considerably between the three datasets (Fig 3); Foothills Forest species achieved the slowest mean maximum relative growth rates of between 0.16 and 0.35 cm cm^-1^ yr^-1^, the Myall Lakes species grew faster than this with rates between 0.39 and 2.10 cm cm^-1^ yr^-1^ while Murray Sunset species grew at between 3.2 and 6.7 cm cm^-1^ yr^-1^ (Fig 3a). In general, species of the Foothill Forests reached their maximum growth significantly later (between 8–21 years) compared to Myall Lakes species (0.68–10 years) and Murray Sunset species (1.1–2.1 years)(Fig 3b). The species from the Foothill forests achieved the greatest heights (between 274–1004 cm), followed by Myall Lake species (20–505 cm) and the shortest were Murray Sunset species (42–463 cm)(Fig 3c).

The Myall Lakes and the Murray Sunset species overlapped greatly in age at which their species reach their maximum growth rates, yet all the Murray Sunset species grew significantly faster than the Myall Lakes species (Fig 3). Maximum growth rate varied greatly between datasets (log grand mean of growth rate Myall Lakes = 0.15, Murray Sunset = 1.35, Foothills = -1.13), but little within datasets (standard deviation: Myall Lakes = 0.321, Murray Sunset = 0.025, Foothills = 0.381). Age at maximum growth varied considerably within datasets (standard deviation: Myall Lakes = 0.741, Murray Sunset = 0.0.118, Foothills = 0.317), and maximum height varies both within (standard deviation: Myall Lakes = 0.719, Murray Sunset = 0.671, Foothills = 0.433) and between datasets.

### Fitting a trait-based model

**Fig 4:**
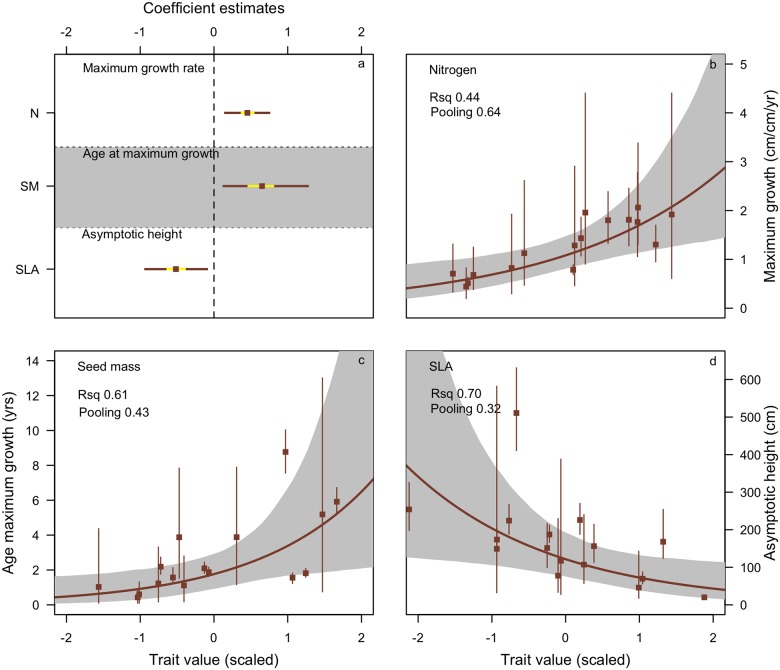
Model fits from the hierarchical growth model to Myall Lakes Species. (a). coefficients for the linear predictors in our growth parameter models. Coefficients describe how traits influence the growth parameter. Positive coefficients indicate a positive relationship between the trait values and the growth parameter. Negative values indicate a negative relationship between the trait and the growth parameter. (b-d) Trait effects on growth parameters. Along the x-axis are the standardized trait values and along the y-axis are the parameter values for each species. Each panel represents a separate trait and that traits effect on that growth parameter. These figures represent partial effect plots, representing the effect of one trait on one growth parameter with all other parameters held at their mean. For example, in the Myall lakes dataset, species with low seed mass reach their maximum growth early, species with higher nitrogen content achieve faster relative growth rates and species with low SLA achieve greater height.

We trained our trait model on the Myall Lakes dataset; which has growth parameter values intermediate to the other two datasets (Fig 3). Within the Myall Lakes dataset, leaf-nitrogen content was significantly positively associated with maximum growth rate, whereby species with high leaf-nitrogen concentrations had faster growth rates than species with lower leaf-nitrogen values. Seed mass was positively and significantly associated with age at maximum growth rate, species with greater seed mass reached their maximum growth later than species with lower seed mass. Specific leaf area (SLA) was significantly negatively associated with maximum height; species with lower SLA achieved greater heights than species with higher SLA (Fig 4).

### Transferring a trait-based model

**Fig 5:**
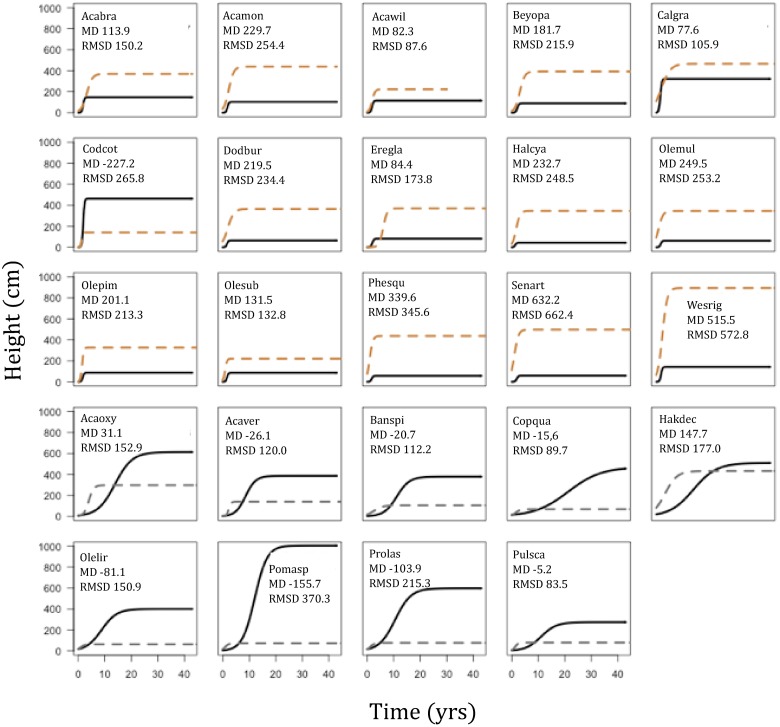
Growth trajectory predictions from the modeled trait relationships from Myall Lakes dataset predicted to Murray Sunset (top three rows) and Foothill forests (bottom two rows) based on species traits in those datasets. All but one of the growth trajectories in Murray Sunset (orange dashed lines) were over predicted compared to modeled trajectories based on Murray Sunset height-data (black solid lines). All the growth trajectories for the Foothill forests (grey dashed lines) were under predicted compared to the trajectories based on the Foothills data (black solid lines). Species names are abbreviated to first three letters of genus and species; in order from left to right: *Acacia brachybotrya*, *Acacia montana*, *Acacia wilhelmiana*, *Beyeria opaca*, *Callitris gracilis*, *Codonocarpus cotinifolius*, *Dodonaea bursarifolia*, *Eremophila glabra*, *Halgania cyanea*, *Olearia muelleri*, *Olearia pimeleoides*, *Phebalium squamulosum*, *Senna artemisioides*, *Westringia rigida*, *Acacia oxycedrus*, *Acacia verticillata*, *Banksia spinulosa*, *Coprosma quadrifida*, *Hakea decurrens*, *Olearia lirata*, *Pomaderris aspera*, *Prostanthera lasianthos*, *Pultenaea scabra*.

The Murray Sunset species were always over predicted by the Myall Lakes model, except one species *Codonocarpus cotinifolius* (Gyrostemonaceae) and the Foothills species are always under predicted (Fig 5). We formalized this obvious visual pattern by calculating the mean deviance and the root mean squared error for each prediction (Fig 5). While some species were predicted quite well—for example the mean deviance in *Acacia wilhelmiana* (Mimosaceae) from Murray Sunset was only 82 cm—most were highly under- or over-predicted and could be up to about 6 m different in height.

The predictive curves were based on trait-growth relationships and the trait values of individual species. When we compared the trait distributions between datasets, we found large differences in the empirical trait distributions, particularly between SLA and leaf-nitrogen (S1 Fig). High SLA values in Murray Sunset National Park were relatively low compared to SLA for species in Myall Lakes; and a low SLA value from the Foothills was average relative to SLA values in Myall Lakes (S1 Fig).

### Do traits play the same role in different ecosystems?

**Fig 6:**
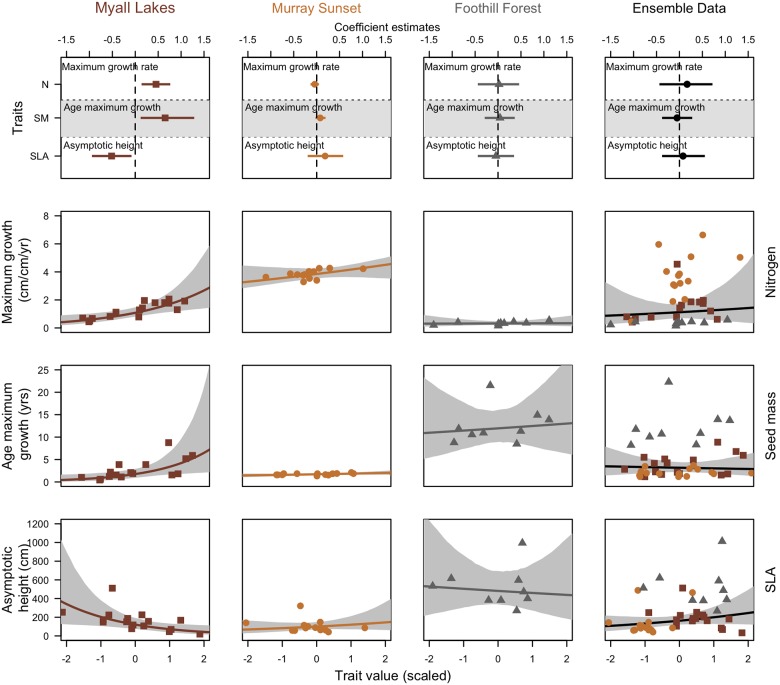
Model fits from the hierarchical growth models to Myall Lakes, Murray Sunset, Foothills and from an ensemble model with those three datasets combined. Positive coefficients indicate a positive relationship between the trait values and growth parameters. Negative values indicate a negative relationship between the trait and the growth parameter. Note: the estimates in the ensemble dataset are re-modeled, so do not directly correspond to the points in each of the individual dataset models.

We tested to see whether the trait-growth influences found in our Myall lakes trait model were found when modeled independently in each of the other two datasets. The traits did not have the same influence on the same growth parameters in each of the datasets (Fig 6). None of the significant trait effects that were apparent in Myall lakes were reflected in the Murray Sunset or the Foothills datasets, with all these relationships mostly being non-significant as well as small and highly uncertain in the case of Foothills (Fig 6). The trends of the relationships between leaf nitrogen and maximum growth rate were positive in each dataset, and for seed mass on the age of maximum growth (Fig 6). Specific leaf area was not consistently related to maximum height across the datasets.

### Does combining datasets improve models?

**Fig 7:**
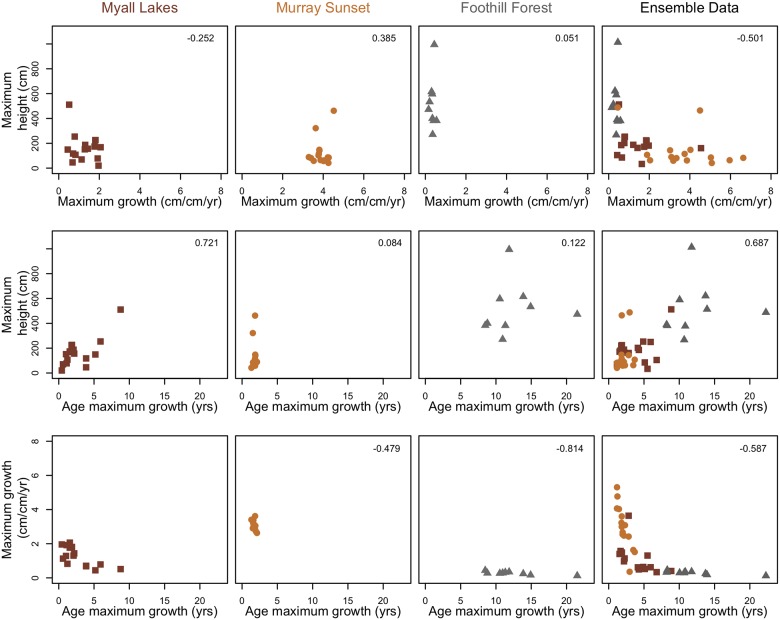
Scatter plots of pairs of each of the model parameters for each dataset and the ensemble dataset. Numbers in the top right hand corner are R^2^ statistics based on Pearson’s correlation coefficient. Brown squares are data from Myall Lakes, tan coloured circles are from Murray Sunset, grey triangles are data from Foothill Forests. Note: the data points in the ensemble dataset are re-modeled, so do not directly correspond to the points in each of the individual dataset models.

When we combined the datasets (Fig 6) and used the ensemble trait distributions we found that the relationships were weaker, all non-significant, relatively uncertain and in the case of seed mass and age of maximum growth (Fig 7) the relationship became negative. Including a varying intercept for sites in this ensemble model, which could possibly account for variation in the mean species slope, had little effect (S2 Fig).

To further investigate the diluted patterns, we looked at the covariance of parameters between each of the datasets and in the ensemble dataset.

The poor transferability of the common trait-based model may be due to different patterns of covariance between model parameters across the vegetation types. Parameters a and b negatively co-varied within each dataset, meaning things that grew fast did so early (Fig 7). Combining the datasets together magnified these patterns. Similarly, in each dataset there was positive covariance between maximum height and age at which maximum growth occurs, so that shorter species reach their maximum growth rates earlier than taller species; this was also magnified in the ensemble dataset (Fig 7). Relationships between maximum height and maximum relative growth rate were weak and inconsistent between regions (negative for Myall Lakes but positive for the other datasets) but became clearly positive in the ensemble dataset. This extra dimension of co-variation that appeared within the ensemble dataset is that species with a greater maximum height (which may occur in different regions) grew more slowly (Fig 7).

## Discussion

One reason generality in ecology is sought, is to enable prediction. In this study we found strong trait-growth relationships in our Myall Lakes dataset; whereby species with low SLA achieved the greatest asymptotic heights, species with high leaf-nitrogen content achieved relatively fast growth rates, and species with low seed mass reached their time of maximum growth early. However these same growth-trait relationships did not hold across the two other datasets, making accurate prediction from one dataset to another unachievable. We suggest there may be (at least) three reasons for this; 1) our models may be overfitted and not represent a systematic or general pattern in the data, 2) height growth trajectories may be fundamentally different between the three ecosystems and 3) trait-growth relationships may change over environmental gradients.

There are some obvious reasons for why trait-based models fitted in one particular area may not be transferable. Over-fitting occurs when a model is highly flexible and fitted so well to the training data that the model captures patterns in the data but also random noise [39,61]. A growth curve constructed like this would have a fabulous fit to one particular data set, but would not be representative of any new or unobserved data. Under-fitting results when important predictor variables, or variables that may explain patterns in the data are ignored and so the model does not capture important biological processes [39,62]. We aimed to balance these concerns by using a data driven statistical model with constrained numbers of parameters and we tried to choose trait-parameter combinations based on findings from the literature. We acknowledge that models are simplistic representations of the world, and that making predictive models in ecology is a difficult task. However, we believe there could be more interesting ecological reasons for why transferring trait-growth models between species and ecosystems is challenging.

There were broad differences in growth parameters between ecosystems. Maximum height values varied widely between and within datasets, but maximum growth rates and the age at which species reach their maximum growth varied more between datasets than within each dataset. Murray Sunset species displayed the fastest growth but lowest overall height whilst species from the Foothills grew the slowest and achieved the tallest heights (Fig 2). We suggest that different ecosystem characteristics lead to different competitive and risk environments after disturbance, which changes the relative importance of height-growth trajectories between ecosystems. There are key structural differences between the canopies of each of the three ecosystems: Foothill Forests and Myall Lakes have taller and more closed canopies dominated by Eucalypts capable of strong epicormic resprouting and generally not fire-killed, whereas Murray Sunset canopy species are basal resprouter mallee eucalypts. These structural differences change the game in terms of the race for light. In Murray Sunset, all species achieve rapid growth, early and there is no distinct temporal partitioning of species dominance. In contrast, Myall Lakes and the Foothill Forests display temporal partitioning, with each species having some time in the sun, which has been reported in other closed canopy systems [18].

It has been suggested there are two axes of variation in plant height, delimited by time since disturbance and vertical light availability, and these may be combined to one dimension of successional growth [18]. Short, fast-growing, early-successional species take advantage of high light environments, whilst taller, slow-growing, late-successional species can compete under shaded conditions. Our study essentially tests this proposition in three different resource–different competitive—environments. Within each independent dataset there was positive co-variation between maximum relative growth rate and the age at which a species reaches maximum growth, therefore plants that grew fast did so early (Fig 7). However, there was no obvious relationship between other pairs of growth parameters–for example within each dataset both tall and short species displayed fast growth. This additional axis of variation, between height and growth rate, only became apparent when the datasets were combined, and it became clear that species that achieve a greater height had a lower maximum relative growth rate (Fig 7). Therefore some broad trends in growth trade-offs are not captured locally within each independent dataset but only become apparent in the ensemble data. Plants that will grow tall do so in productive environments, but presumably because they need to support themselves at greater heights (for longer) do not grow so quickly. Within the ensemble data not only did co-variation in growth parameters change, but the trait-growth relationships became weak, non-significant and uncertain (Fig 6). This was not aided by adding site as random intercepts to the trait sub-models (S2 Fig), suggesting that ensemble modeling of multi-species from different environments can dilute important trait-growth effects that may occur between some species in some environments but not others.

Our three datasets contained species with different and sometimes non-overlapping trait values (S2 Fig), with Murray Sunset species having significantly lower SLA values than species in Myall Lakes and the Foothills. Weak predictions based on functional traits have been found in communities where there was low variation in functional traits [28] and it has been demonstrated that while sample size and sampled trait range affect relationships between leaf traits within sites, general, though not universal, patterns in trait relationships exist at different sites [32]. The different magnitudes of trait values may make it difficult to predict from one system to another, and mean that when these data are combined in an ensemble dataset the increased variation may not correspond to the same variation in growth between ecosystems.

Another explanation for poor trait-growth predictability across ecosystems, is that the importance of traits influencing growth strategies might not hold in different environments. We found that the trait-growth relationships were not consistent between ecosystems. Many trait combinations occur among species [18], and a variety of strategies can sustain viable populations [18,31]. Particular trait combinations maybe selected for because they give a competitive advantage in one environment compared to other possible combinations. Ambiguous relationships are found in the literature between growth rates and functional traits with some authors suggesting we have little capacity to generalize based on traits [63], particularly at global scales [4, 28]. An alternative explanation is that there could be real species-growth-trait differences that are driven by productivity between ecosystems and that we have not been successful at disentangling yet.

All studies are limited by with sample size. Our study is limited by the numbers of species within regions (limiting the inference about trait effects on growth parameters), as well as the number of regions representing different ecosystems (limiting inference about how trait effects might vary between regions). Ideally, one would like to have datasets containing as many species as possible covering a wide trait distribution that have been structurally sampled across a productivity gradient. If datasets from several ecosystems were available, environmental covariates could be explicitly incorporated into our hierarchical models. There are many growth models to choose from, and as we have discussed, it remains to be seen whether sub-optimal model fits are due to dataset size, or deeper underlying biological reasons. An important area of future research is interrogating many different growth models across different datasets to further diagnose model behaviors as well as to support the ongoing process of finding robust and biologically relevant model evaluation tools.

That plants grow differently in different environments is well appreciated. However, how height-growth trajectories of multiple species systematically change over environmental gradients and how this affects our ability to generalize demographic rates across species and ecosystems is not clear. Furthermore, it seems likely that there is a complex interplay between species functional traits and their height-growth trajectories that is not yet well understood. This has major implications if our aim is to build predictive models that can be used to extend hypotheses or to make broad generalizations. A future area for research is to disentangle site-specific trait correlations from systematic effects, to try to understand whether the trait-growth relationships we discover result from functional reasons or from correlations among traits that are unrelated to growth. That is, we need to tease apart causal inferences from correlated inference. Apart from being a fundamental goal of much ecological research, finding generality could have enormous practical importance for making management decisions, and this will be most relevant at local scales.

## Supporting information

S1 Fig Raw trait value scatterplots compared between each trait in each ecosystem. Numbers in the top right hand corner are R^2^ statistics based on Pearson’s correlation coefficient. Brown squares are data from Myall Lakes, sand coloured circles are from Murray Sunset, grey triangles are data from Foothill Forests and black circles are the combined ensemble data.
(TIF)

S2 Fig Model fits from the hierarchical growth model with the ensemble dataset where vegetation type is included as a varying intercept. Brown squares are data from Myall Lakes, sand coloured circles are from Murray Sunset National Park, grey triangles are data from Foothill Forests.
(TIF)

S3 Fig The relationships between the maximum growth rate parameter of the Hillslope equation with units cm cm^-1^ yr^-1^ compared to derived growth rates of absolute growth rate and relative growth rate. Numbers in the top right hand corner are R^2^ based on Pearson’s correlation coefficient. Brown squares are data from Myall Lakes, sand coloured circles are from Murray Sunset and grey triangles are data from Foothill Forests.
(TIF)

S4 Fig The relationships between the maximum growth rate parameter of the Hillslope equation, absolute growth rate (Max AGR) and relative growth rate (Max RGR) and functional traits. Numbers in the top right hand corner are R^2^ based on Pearson’s correlation coefficient. These data are for the Murray Sunset dataset.
(TIF)

S5 Fig The relationships between the maximum growth rate parameter of the Hillslope equation, absolute growth rate and relative growth rate and functional traits. Numbers in the top right hand corner are R^2^ based on Pearson’s correlation coefficient. These data are for the Myall Lakes dataset.
(TIF)

S6 Fig The relationships between the maximum growth rate parameter of the Hillslope equation, absolute growth rate and relative growth rate and functional traits. Numbers in the top right hand corner are R^2^ based on Pearson’s correlation coefficient. These data are for the Foothill Forest dataset.
(TIF)

S1 File Description of field sampling in Murray Sunset National Park. (DOCX)

## References

[1] Beven K. Towards a coherent philosophy for modeling the environment. Proc. R. Soc. London, Ser. A. 2002; 458: 2465–2484.

[2] Harris GP. Pattern, process and prediction in aquatic ecology. A limnological view of some general ecological problems. Freshw. Biol. 1994; 32: 143–160.

[3] Westoby M, Falster DS, Moles AT, Vesk PA, Wright IJ. Plant ecological strategies: some leading dimensions of variation between species. Annu. Rev. Ecol. Syst. 2002; 33: 125–159.

[4] Paine CET, Amissah L, Auge H, Baraloto C, Baruffol M, Bourland N, et al Globally, functional traits are weak predictors of juvenile tree growth, and we do not know why. J. Ecol. 2015; 103: 978–989.

[5] Sandel B, Corbin JD, Krupa M. Using plant functional traits to guide restoration: A case study in California coastal grassland. Ecosphere. 2011; 2: 1–16.

[6] May FE, Giladi I, Ristow M, Ziv Y, Jeltsch. Plant functional traits and community assembly along interacting gradients of productivity and fragmentation. Perspect. Plant Ecol. Evol. Syst. 2013; 15: 304–318.

[7] Martinez-Garza C, Bongers F, Poorter L. Are functional traits good predictors of species performance in restoration plantings in tropical abandoned pastures? For. Ecol. Manage. 2013; 303: 35–45.

[8] Pickering CM, Venn SE. Increasing the resilience of the Australian alpine flora to climate change and associated threats: a plant functional traits approach. National Climate Change Adaptation Research Facility, Gold Coast pp.94 2013.

[9] Vesk PA, Westoby M. Predicting plant species’ responses to grazing. Appl. Ecol. 2001; 38: 897–909.

[10] Diaz S, Lavorel S, McIntyre S, Falczuk V, Casanoves F, Michunas DG, et al Plant trait responses to grazing–a global synthesis. Glob. Chang. Biol. 2007; 13: 313–341.

[11] Herault B, Ouallet J, Blanc L, Wagner F, Baraloto C. Growth responses of neotropical trees to logging gaps. J. Appl. Ecol. 2010; 47: 821–831.

[12] Schwilk DW, Caprio AC. Scaling from leaf traits to fire behavior: community composition predicts fire severity in temperate forest. J. Ecol. 2011; 99: 970–980.

[13] Mayfield MM, Dwyer JM, Chalmandier L, Wells JA, Bonser SP, Catterall CP, et al Differences in forest plant functional trait distributions across land-use and productivity gradients. Am. J. Bot. 2013; 100: 1356–1368. 10.3732/ajb.1200461 23825137

[14] Williams NSG, Hahs AK, Vesk PA. Urbanisation, plant traits and the composition of urban floras. Perspect. Plant Ecol. Evol. Syst. 2015; 17: 78–86.

[15] Kitajima K. Relative importance of photosynthetic traits and allocation patterns as correlates of seedling shape tolerance of 13 tropical trees. Oecologia. 1994; 98: 419–428. 10.1007/BF00324232 28313920

[16] Kobe R. Light gradient partitioning among tropical tree species through differential seedling mortality and growth. Ecology. 1999; 80: 187–201.

[17] Thomas SC, Bazzaz FA. Asymptotic height as a predictor of photosynthetic characteristics in Malaysian rain forest trees. Ecology. 1999; 80: 1607–1622.

[18] Falster DS, Westoby M. Alternative height strategies among 45 dicot rain forest species from tropical Queensland, Australia. J. Ecol. 2005b; 93: 521–535.

[19] King DA, Davies SJ, Nur Supardi MN, Tan S. Tree growth is related to light interception and wood density in two mixed dipterocarp forests of Malaysia. Funct. Ecol. 2005; 19: 445–453.

[20] Sterk FJ, Poorter L, Schieving F. Leaf traits determine the growth-survival trade-off across rain forest tree species. Am. Nat. 2006; 167: 758–765. 10.1086/503056 16671019

[21] Poorter L, Wright SJ, Paz H, Ackerly DD, Condit R, Ibarra-Manriquez G, et al Are functional traits good predictors of demographic rates? Evidence from five Neotropical forests. Ecology. 2008; 89: 1908–1920. 18705377 10.1890/07-0207.1

[22] Martinez-Vilalta J, Mencuccini M, Vayreda J, Retana J. Interspecific variation in functional traits, not climatic differences among species ranges, determines demographic rates across 44 temperate and Mediterranean tree species. J. Ecol. 2010; 98: 1462–1475.

[23] Ruger N, Condit R. Testing metabolic theory with models of tree growth that include light competition. Funct. Ecol. 2012; 26: 759–765.

[24] Adler PB, Salguero-Gomez R, Compagnoni A, Hsu JS, Ray-Mukherjee J, Mbeau-Ache C, et al Functional traits explain variation in plant life history strategies. Proc. Natl. Acad. Sci. 2014; 111: 740–745. 10.1073/pnas.1315179111 24379395 PMC3896207

[25] Kunstler G, Falster D, Coomes DD, Hui F, Kooyman RM, Laughling DC, et al (2016) Plant functional traits have globally consistent effects on competition. Nature. 2016; 204–207. 10.1038/nature16476 26700807

[26] Diaz S, Kattge J, Cornelissen JHC, Wright IJ, Lavorel S, Dray S. et al The global spectrum of plant form and function. Nature. 2015; 1–5. 10.1038/nature16489 26700811

[27] Cordlandwehr V, Meredith RL, Ozinga WA, Bekker RM, van Groenendael JM, Bakker JP. Do plant traits retrieved from a database accurately predict on-site measurements? J. Ecol. 2013; 101: 662–670.

[28] Funk JL, Cornwell WK. Leaf traits within communities: Context may affect the mapping of traits to function. Ecology. 2013; 94(9): 1893–1897. 24279259 10.1890/12-1602.1

[29] Reich PB. The world-wide “fast-slow” plant economics spectrum: a traits manifesto. J. Ecol. 2014; 102: 275–301

[30] Cunningham SA, Summerhayes B, Westoby M. Evolutionary divergences in leaf structure and chemistry, comparing rainfall and soil nutrient gradients. Ecology. 1999; 69: 569–588.

[31] Fonesca CR, Overton JM, Collins B, Westoby M. (2000) Shifts in trait-combinations along rainfall and phosphorus gradients. J. Ecol. 2000; 88: 964–977.

[32] Wright IJ, Reich PB, Westoby M. Strategy shifts in leaf physiology, structure and nutrient content between species of high- and low-rainfall and high- and low-nutrient habitats. Funct. Ecol. 2001; 15: 423–434.

[33] Wright IJ, Reich PB, Cornelissen JHC, Falster DS, Garnier E, Hikosaka K, et al Assessing the generality of global leaf trait relationships. New Phytol. 2005; 166: 485–496. 10.1111/j.1469-8137.2005.01349.x 15819912

[34] Wilfahrt PA, Collins B, White PS. Shifts in functional traits among tree communities across succession in eastern deciduous forests. For. Ecol. Manage. 2014; 1–7.

[35] Falster DS, Moles AT, Westoby M. A general model for the scaling of offspring size and adult size. Am. Nat. 2008; 172(3): 299–317. 10.1086/589889 18631112

[36] Hérault B, Bachelot B, Poorter L, Rossi V, Bongers F, Chave J, et al Functional traits shape ontogenetic growth trajectories of rain forest tree species. J. Ecol. 2011; 99: 1431–1440.

[37] Randin CF, Dirnbock T, Dullinger S, Zimmerman NE, Zappa M, Guisan A. Are niche-based species distribution models transferable in space? J. Biogeogr. 2006; 33: 1689–1703.

[38] Elith J, Leathwick JR. Species distribution models: ecological explanation and prediction across space and time. Annu. Rev. Ecol. Evol. Syst. 2009; 40: 677–697.

[39] Wenger SJ, Olden JD. Assessing transferability of ecological models: an underappreciated aspect of statistical validation. Methods Ecol. Evol. 2012; 3: 260–267.

[40] Hooten MB, Hobbs NT. A guide to Bayesian model selection for ecologists. Ecol. Monogr. 2015; 85: 3–28.

[41] Cornelissen J, Lavorel S, Garnier E, Diaz S, Buchmann N, Gurvich DE et al A handbook of protocols for standardised and easy measurement of plant functional traits worldwide. Aust. J. Bot. 2003; 51: 335–380.

[42] Specht RL. Vegetation In Australian Environment 4th edn (ed Leeper G.W), Melbourne Univ. Press; 1970 p. 44–67.

[43] Falster DS, Westoby M. Tradeoffs between height growth rate, stem persistence and maximum height among plant species in a post-fire succession. Oikos. 2005a; 111: 57–66

[44] Muir AM, Vesk PA, Hepworth G. Reproductive trajectories over decadal time-spans after fire for eight obligate-seeder shrub species in south-eastern Australia. Aust. J. Bot. 2014; 62: 369–10.

[45] Limpert E, Stahel WA, Abbt M. Log-normal Distributions across the Sciences: Keys and Clues. Bioscience. 2001; 51: 341–352.

[46] Tjørve E. Shapes and functions of species–area curves: a review of possible models. J. Biogeogr. 2003; 30: 827–835.

[47] Tjørve E, Tjørve KMC. A unified approach to the Richards-model family for use in growth analyses Why we need only two model forms. J. Theor. Biol. 2010; 267: 417–425. 10.1016/j.jtbi.2010.09.008 20831877

[48] Paine CET, Marthews TR, Vogt DR, Purves D, Rees M, Hector A, et al How to fit nonlinear plant growth models and calculate growth rates: an update for ecologists. Methods Ecol. Evol. 2012; 3: 245–256.

[49] Tjørve E. Shapes and functions of species-area curves (II): a review of new models and parameterizations. J. Biogeogr. 2009; 36: 1435–1445.

[50] Moles AT, Westoby M. Seed size and plant strategy across the whole life cycle. Oikos. 2006; 113: 91–105.

[51] Thomas SC. Asymptotic height as a predictor of growth and allometric characteristics in Malaysian rain forest trees. Am. J. Bot. 1996; 83: 556–566.

[52] Plummer M. JAGS Version 3.3. 0 user manual. 2011. http://www.tp.iweb.dl.sourceforge.net/project/mcmc-jags/Manuals/3.x/jags_user_manual.pdf.

[53] Su YS, Yajima M. SystemRequirements, J. A. G. S. Package ‘R2jags’. 2015.

[54] Gelman A. Prior distributions for variance parameters in hierarchical models (comment on article by Browne and Draper). Bayesian Anal. 2006; 3: 515–534.

[55] Brooks SP, Gelman A. General methods for monitoring convergence of iterative simulations. J. Comput. Graph. Stat. 1998; 7: 434–455.

[56] Spiegelhalter DJ, Best NG, Carlin BP, van der Linde A. Bayesian measures of model complexity and fit. J. R. Stat. Soc. Series B Stat. Methodol. 2002; 64: 583–639.

[57] Gelman A, Pardoe I. Bayesian Measures of Explained Variance and Pooling in Multilevel (Hierarchical) Models. Technometrics. 2006; 48: 241–251.

[58] Cumming G, Finch S. Inference by eye: confidence intervals and how to read pictures of data. Am. Psychol. 2005; 60: 170–180. 10.1037/0003-066X.60.2.170 15740449

[59] Kobayashi K, Salam MS. Comparing simulated and measured values using mean squared deviation and its components. Agron. J. 2000; 92: 345–352.

[60] Walther BA, Moore JL, Rahbek C. The concepts of bias, precision and accuracy, and their use in testing the performance of species richness estimators, with a literature review of estimator performance. Ecography. 2005; 28: 815–829.

[61] Olden JD, Jackson DA. Torturing data for the sake of generality: How valid are our regression models? Ecoscience. 2000; 7: 501–510.

[62] Justice AC, Covinsky KE, Berlin JA. Assessing the generalizability of prognostic information. Ann. Intern. Med. 1999; 130: 515–524. 10075620 10.7326/0003-4819-130-6-199903160-00016

[63] Mlambo MC. Not all traits are “functional”: insights from taxonomy and biodiversity-ecosystem functioning research. Biodivers. Conserv. 2014; 23: 781–790.

