# Supplementary figures and images for: Are trait-growth models transferable? Predicting multi-species growth trajectories between ecosystems using plant functional traits
Source: PLoS One. 2017 May 9;12(5):e0176959. doi: 10.1371/journal.pone.0176959 (PMC5423618; doi:10.1371/journal.pone.0176959)

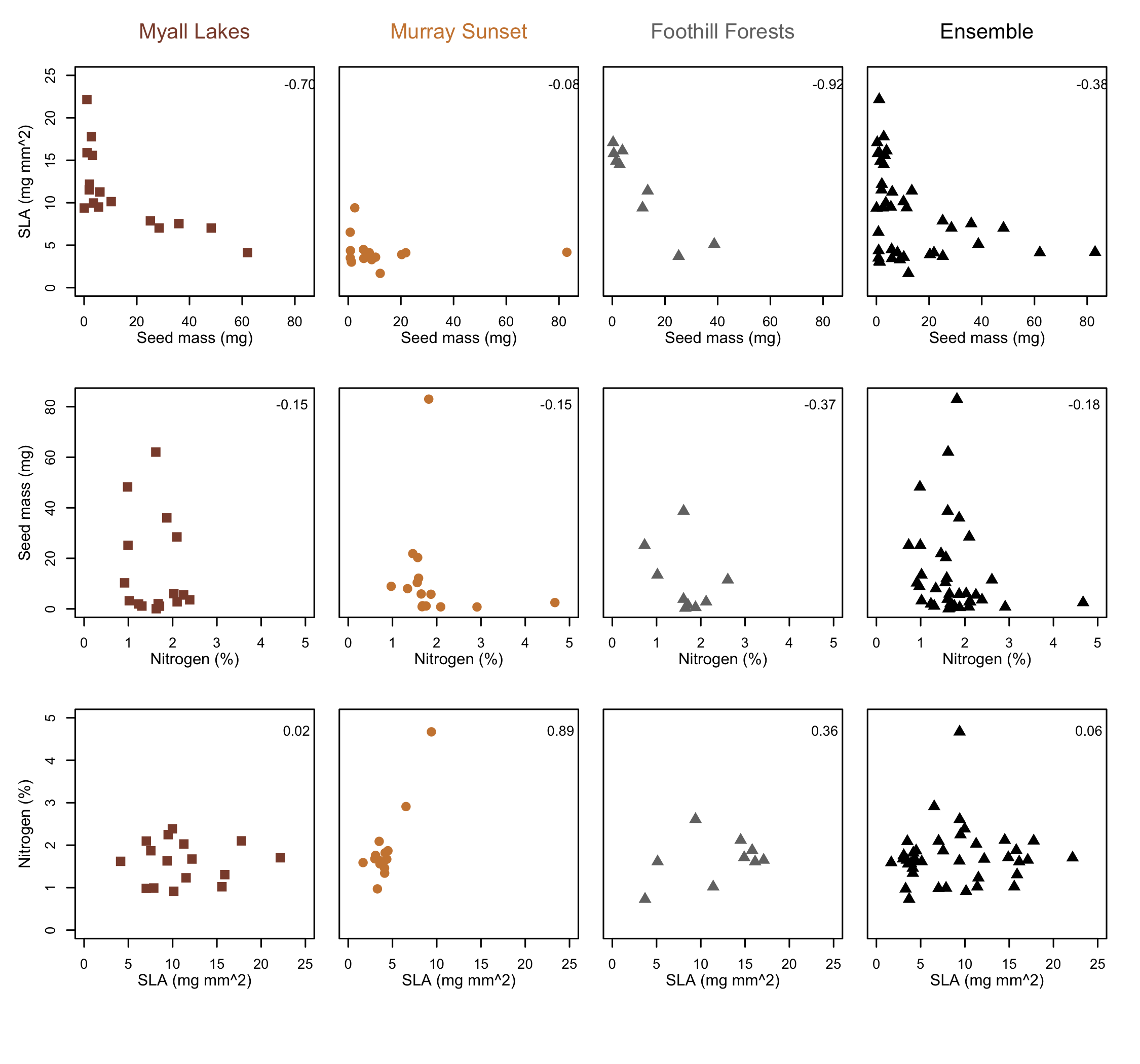

Supplement: S1 Fig — Numbers in the top right hand corner are R2 statistics based on Pearson’s correlation coefficient. Brown squares are data from Myall Lakes, sand coloured circles are from Murray Sunset, grey triangles are data from Foothill Forests and black circles are the combined ensemble data. (TIF) [file pone.0176959.s001.tif]

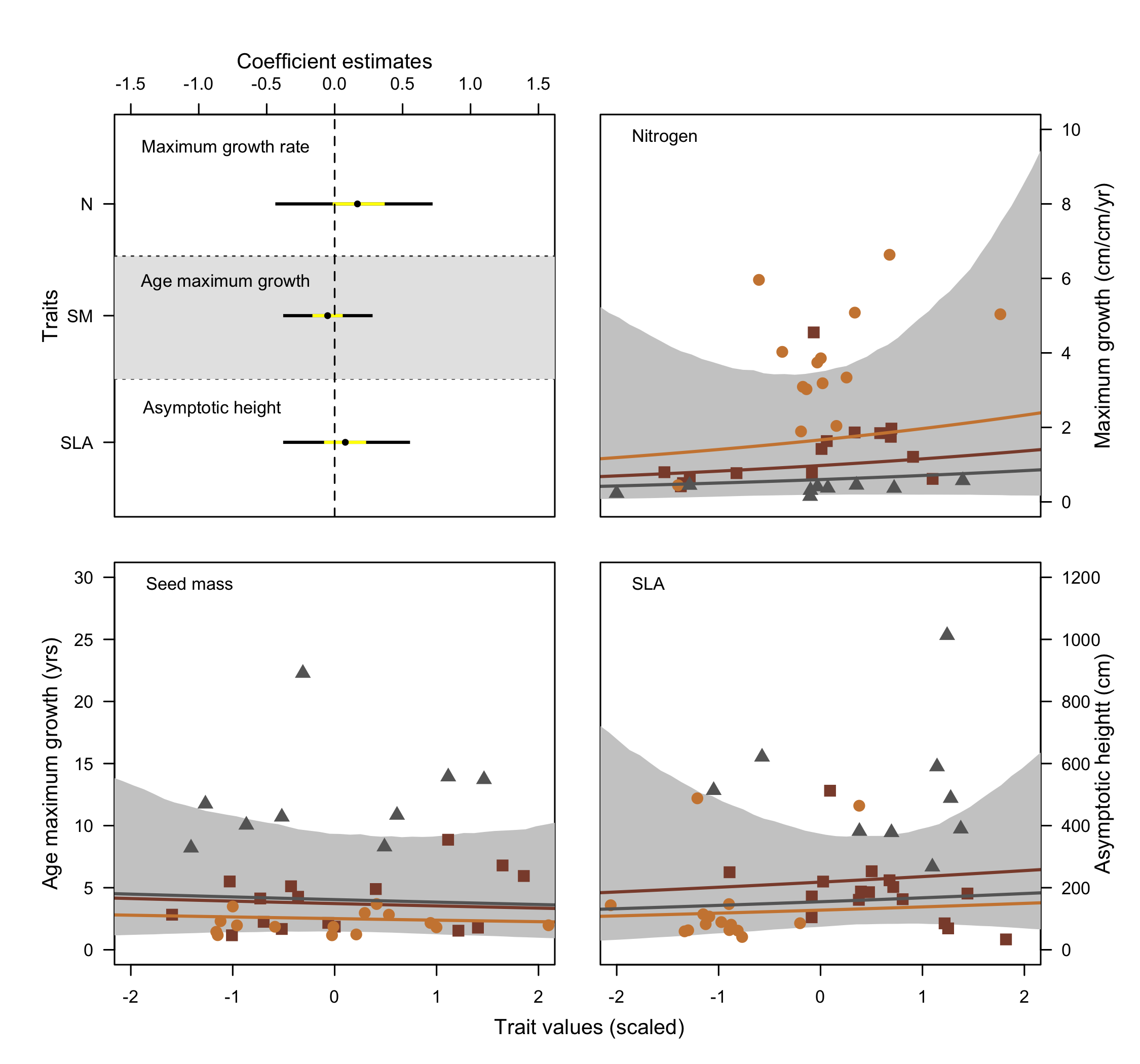

Supplement: S2 Fig — Brown squares are data from Myall Lakes, sand coloured circles are from Murray Sunset National Park, grey triangles are data from Foothill Forests. (TIF) [file pone.0176959.s002.tif]

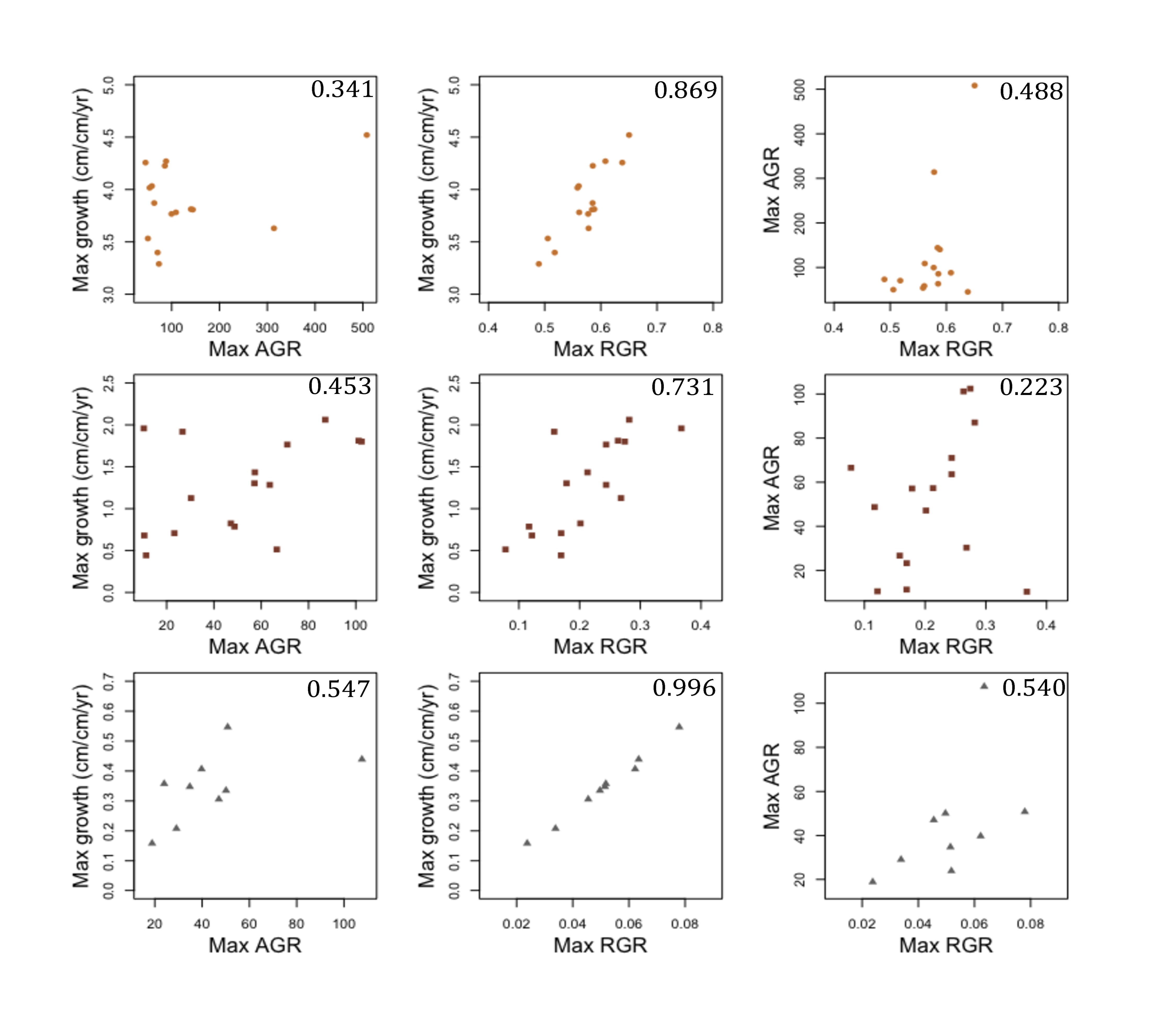

Supplement: S3 Fig — Numbers in the top right hand corner are R2 based on Pearson’s correlation coefficient. Brown squares are data from Myall Lakes, sand coloured circles are from Murray Sunset and grey triangles are data from Foothill Forests. (TIF) [file pone.0176959.s003.tif]

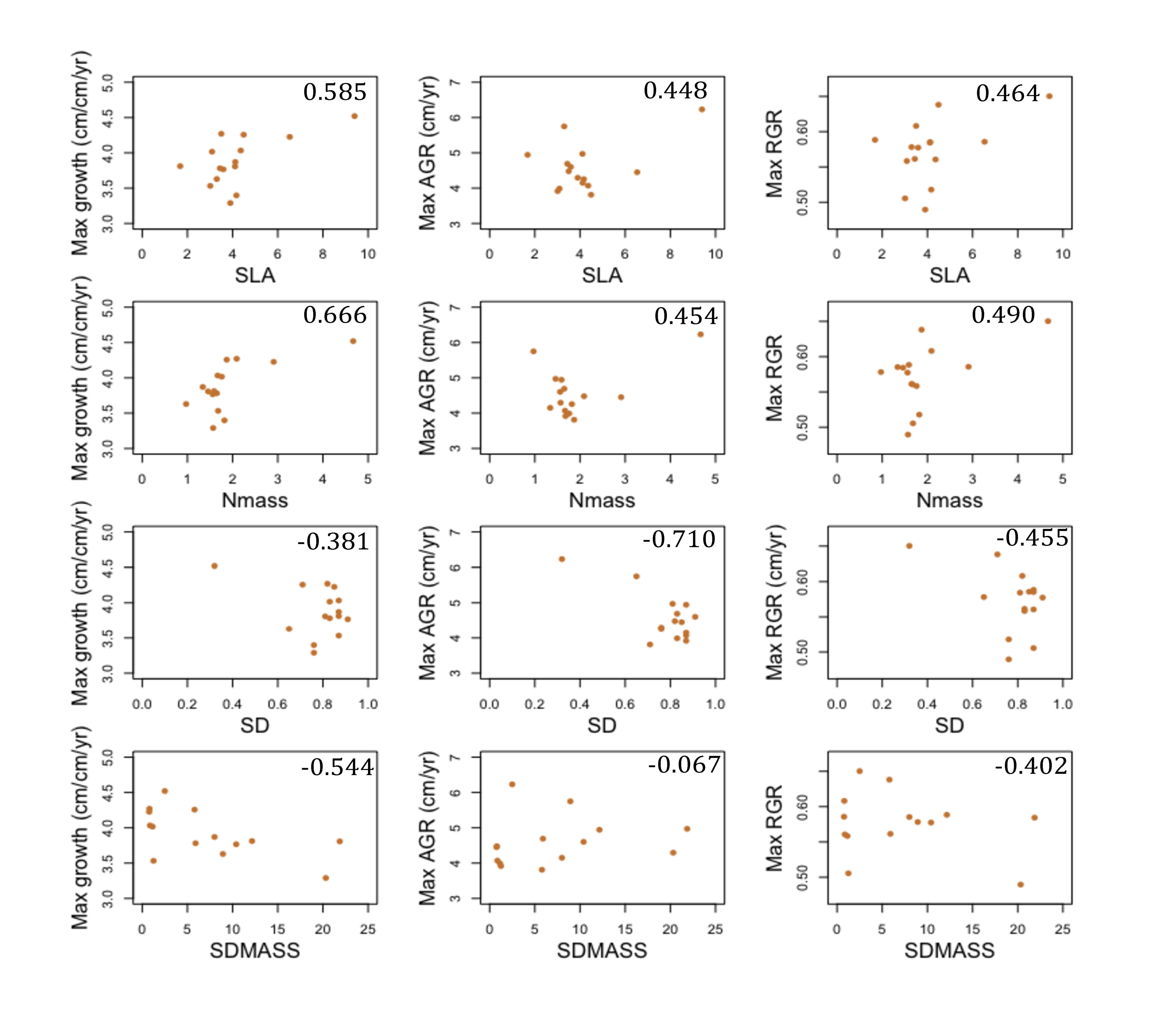

Supplement: S4 Fig — Numbers in the top right hand corner are R2 based on Pearson’s correlation coefficient. These data are for the Murray Sunset dataset. (TIF) [file pone.0176959.s004.tif]

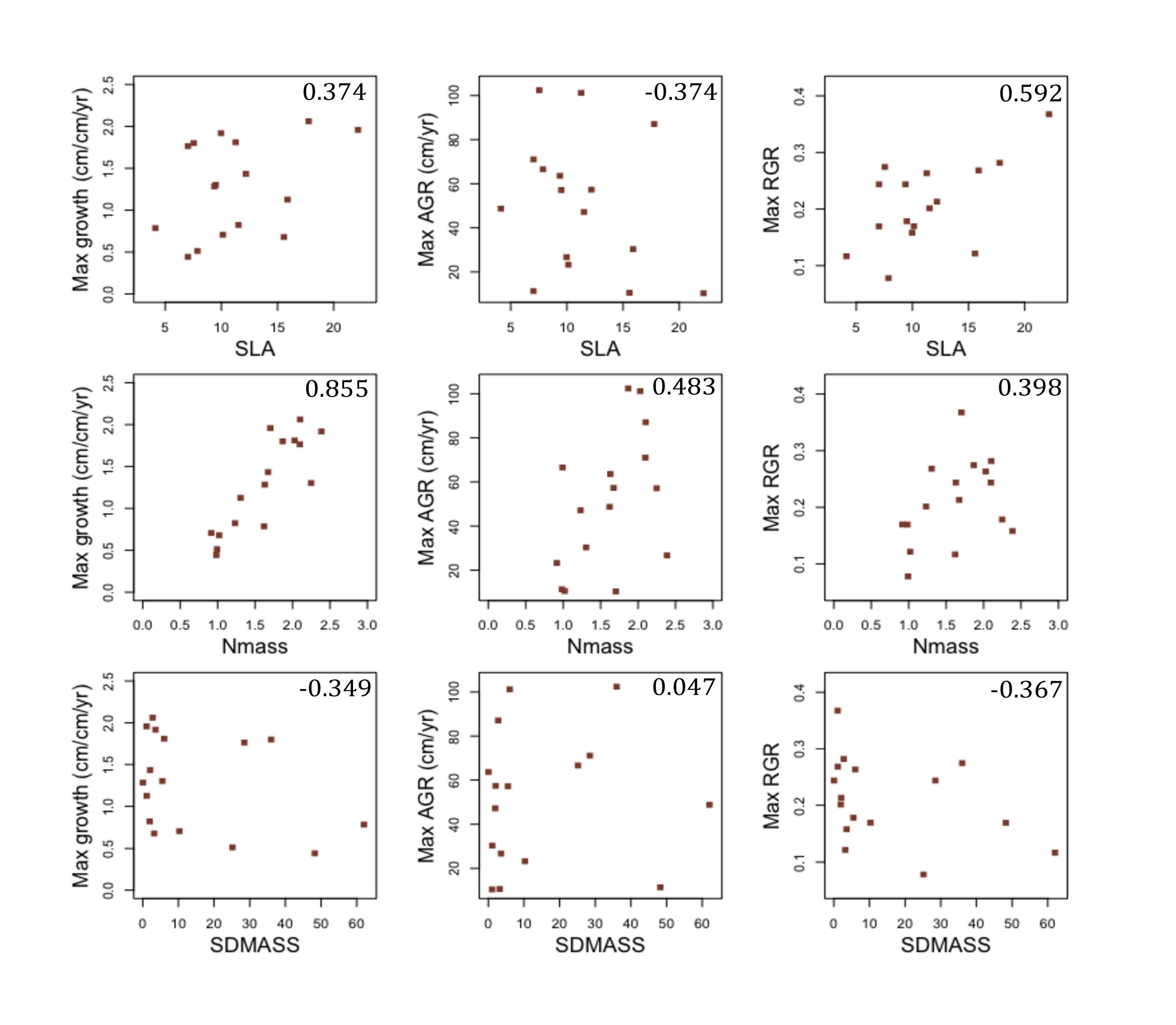

Supplement: S5 Fig — Numbers in the top right hand corner are R2 based on Pearson’s correlation coefficient. These data are for the Myall Lakes dataset. (TIF) [file pone.0176959.s005.tif]

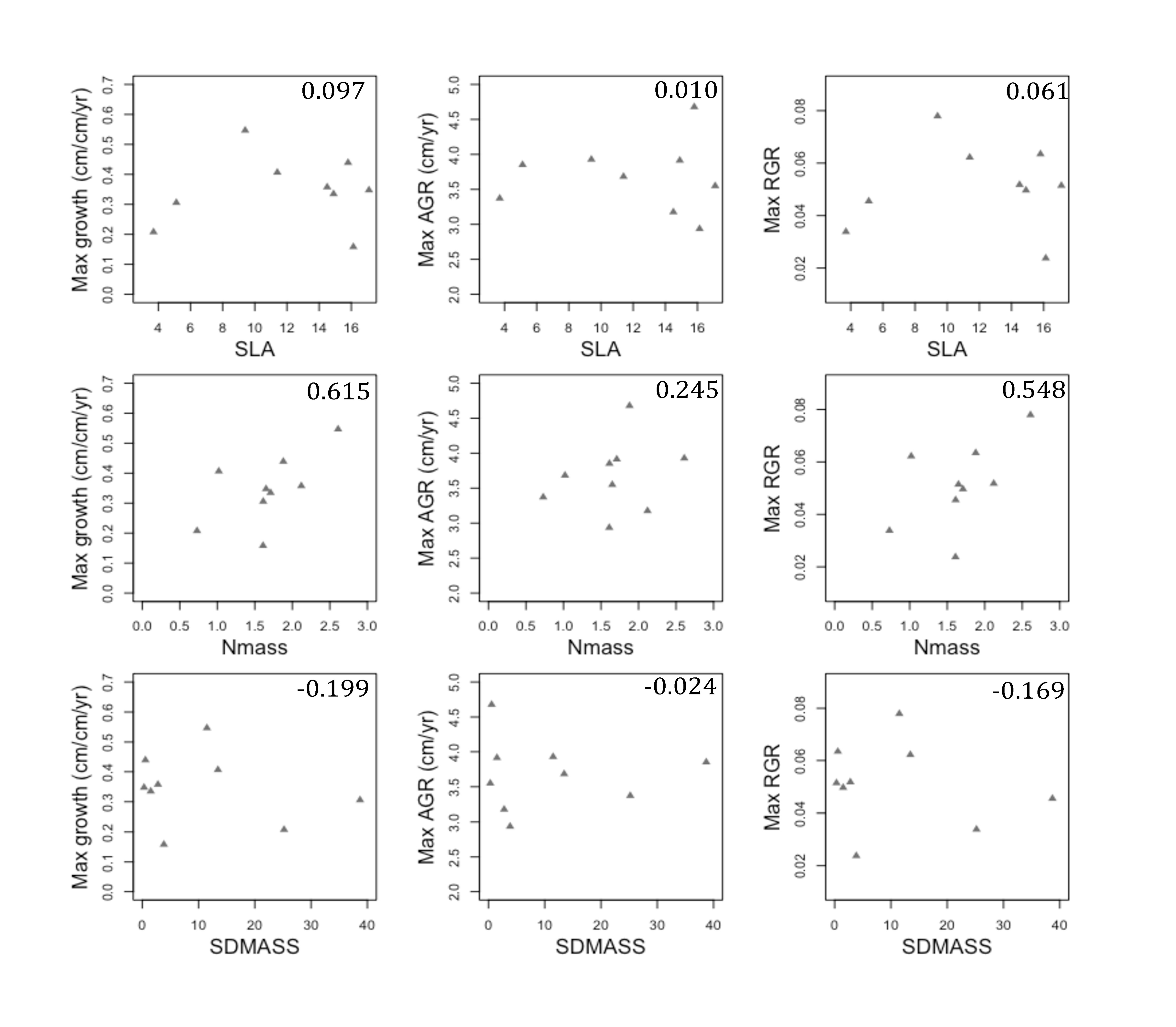

Supplement: S6 Fig — Numbers in the top right hand corner are R2 based on Pearson’s correlation coefficient. These data are for the Foothill Forest dataset. (TIF) [file pone.0176959.s006.tif]
