# Supplementary material for: Are trait-growth models transferable? Predicting multi-species growth trajectories between ecosystems using plant functional traits
Source: PLoS One. 2017 May 9;12(5):e0176959. doi: 10.1371/journal.pone.0176959 (PMC5423618; doi:10.1371/journal.pone.0176959)
Supplement: S1 File — (DOCX) [file pone.0176959.s007.docx]

**Methods**

*Field sampling in Murray Sunset National Park, Victoria*

Heights of at least five (and up to 10) individuals per species were measured in each of eleven time-since-fire areas. Guidance on sample sizes for measuring plant traits were followed from Cornellissen *et al.* (2003). We took five samples from each of five individual plants for each species. Specific leaf area (SLA; mg mm2), the one-sided leaf surface area of fresh leaves divided by the mass of leaves oven-dried at 65°C for 48 h was measured for at least five leaves from each of five individuals for each species. Leaf nitrogen concentrations (Nmass; %) were calculated on five fully expanded leaves taken from well-lit positions on each of five individuals per species. Leaves from each species were then pooled and finely ground for nitrogen analysis. Total nitrogen concentration based on mass (%) was measured using complete combustion gas chromatography performed by The Surface and Chemical Analysis Network at The University of Melbourne. Stem tissue density (dry mass per unit fresh volume; mg mm^-3^) was measured using 40-60 mm long, stem segments from at least five individuals per species. Samples were collected in the field and refrigerated before processing as soon as feasible. Stem tissue density was then determined following Archimedes principle using the protocol from Cornellissen *et al.* (2003). Seed mass (mg) was measured for as many species as possible with field-collected seed, oven-dried and weighed. Seed mass data was also supplemented from the literature and a global seed mass database (Moles & Westoby 2003). Field permit from The Department of Environment, Land, Water and Planning.
